# Development of an *in Silico* Model of DPPH• Free Radical Scavenging Capacity: Prediction of Antioxidant Activity of Coumarin Type Compounds

**DOI:** 10.3390/ijms17060881

**Published:** 2016-06-07

**Authors:** Elizabeth Goya Jorge, Anita Maria Rayar, Stephen J. Barigye, María Elisa Jorge Rodríguez, Maité Sylla-Iyarreta Veitía

**Affiliations:** 1 Pharmacy Department, Faculty of Chemistry and Pharmacy, Central University “Marta Abreu” of Las Villas, C-54830 Santa Clara, Cuba; egoyaj@gmail.com or egoyaj@yandex.com (E.G.J.); elisajorge@yahoo.es (M.E.J.R.); 2 Equipe de Chimie Moléculaire du Laboratoire CMGPCE, EA 7341, Conservatoire National des Arts et Métiers, 2 rue Conté, 75003 Paris, France; anitarayar@hotmail.fr; 3 Department of Chemistry, Federal University of Lavras, P.O. Box 3037, 37200-000 Lavras, Brazil; sjbarigye@gmail.com

**Keywords:** artificial neural networks, MLP, antioxidant, QSAR, DPPH•, free radical scavenger, coumarin

## Abstract

A quantitative structure-activity relationship (QSAR) study of the 2,2-diphenyl-l-picrylhydrazyl (DPPH•) radical scavenging ability of 1373 chemical compounds, using DRAGON molecular descriptors (MD) and the neural network technique, a technique based on the multilayer multilayer perceptron (MLP), was developed. The built model demonstrated a satisfactory performance for the training $\left(R^{2}=0.713\right)$ and test set $\left(Q_{\text{ext}}^{2}=0.654\right)$, respectively. To gain greater insight on the relevance of the MD contained in the MLP model, sensitivity and principal component analyses were performed. Moreover, structural and mechanistic interpretation was carried out to comprehend the relationship of the variables in the model with the modeled property. The constructed MLP model was employed to predict the radical scavenging ability for a group of coumarin-type compounds. Finally, in order to validate the model’s predictions, an *in vitro* assay for one of the compounds (4-hydroxycoumarin) was performed, showing a satisfactory proximity between the experimental and predicted pIC_50_ values.

## 1. Introduction

The discovery of antioxidant agents has attracted much attention in recent years, because oxidative damage is related to many pathological conditions [1]. Compounds whose antioxidant activity is based on scavenge free radicals are among the most important and studied antioxidants. Numerous *in vivo* and *in vitro* methods have been developed to measure the antioxidant capacity and effectiveness, but there is no universal technique by which this property can be estimated precisely and quantitatively [2,3,4,5]. The *in vitro* scavenging capacity can be evaluating using biological oxidants such as nitric oxide radical (*NO*•) and hydroxyl radical (*OH*•), or with nonbiological oxidants such as 2,2′-azino-bis-(3-ethylbenzothiazoline-6-sulphonate) radical cation (ABTS) and 2,2-diphenyl-1-picrylhydrazyl radical (DPPH•). The capturing of the DPPH• radical is one of the best-known methods [2,6,7].

A great number of coumarin derivatives have been studied for their biochemical and pharmacological profiles. Some studies suggest that these compounds may significantly affect the function of various mammalian cellular systems. Specifically, their antioxidant effect has been explored, since the structural features of this group of compounds suggest that they can exhibit this pharmacologic property [8,9,10].

Chemoinformatics tools are used in the modeling of the antiradical activity, as well as other biological properties, given their advantages in saving time and resources [11,12]. Complex associations between biological activities and chemical features of compounds have been approached with mathematical models of quantitative structure-activity relationships (QSAR). [13]. Several statistical and machine learning methods have been widely used in the literature to build models for the QSAR study of chemical compounds. For the last two decades, artificial neural network (ANN) techniques have increasingly found applicability in QSAR studies, thanks to their ability to map non-linear relations between structural characteristics of chemical compounds and their chemical/biological behavior [14].

The objective of this study was to develop an ANN model in order to relate the chemical compounds’ scavenging ability of the DPPH• radical with the corresponding structural features, also known as molecular descriptors (MD), in a set of 1373 molecules. Then, the built model was used to predict the antioxidant activity of a group of coumarin derivatives and an *in vitro* study of 4-hydroxycoumarin was performed to corroborate the result predicted by the network.

## 2. Results

### 2.1. Modeling

Compound*s*: The whole dataset of 1373 chemical compounds was optimized for three-dimensional (3D) coordinates with CORINA software (Molecular Networks GmbH: Erlangen, Germany) [15], a process that displayed 13 molecules as structural outliers. For building the model, the dataset was divided into training and test sets, comprised of 1017 and 339 compounds, respectively. Four compounds were excluded as they showed atypical behavior in the clusters division. From the amalgamation schedule of the hierarchical cluster analysis, nine clusters were determined and posteriorly used in the K-means clustering experiment where 25% of the compounds from each cluster were used as the test set.

Molecular descriptors selection: The x/x correlation filter (based on a correlation coefficient threshold of 0.90) and the standardized entropy measure (less than 0.30) available in the MobyDigs software (TALETE srl., Milano, Italy) [16] allowed for the reduction of the original data matrix to 478 MD. Posteriorly, supervised feature selection based on multiple linear regression coupled with the genetic algorithm was employed to select 14 MD, considered as the most significant for the modeling of DPPH• free radical scavenging capacity, *i.e.*, MATS2e, BELe6, HATS3u, H2v, R7v, nN-N, nImidazoles, C-005, C-020, O-057, O-060, GVWAI-50, B02 (O-S) and B07 (O-S).

QSAR method: The multilayer perceptron (MLP) neural model was constructed using the DPPH• scavenging capacity of 1356 molecules. Outlier diagnosis was performed using the William’s plot where data points with residuals greater than two standard deviation units were deemed to be outliers. Additionally, an analysis of the leverage values was performed to avoid the model’s dependence on particular data points. Therefore, 31 compounds were separated from the model and the network was finally constructed with 1325 molecules. Several options were explored, giving, as the best final configuration of the network, the one based on the Quasi-Newton method as the training algorithm: Broyden-Fletcher-Goldfarb-Shanno [17], with 90 interaction cycles (BFGS 90); the error function was the sum-of-squares (SOS), and Tanh and Logistic were employed as activation functions used for the hidden and output layers, respectively. The 14 MD selected as the most significant variables were used as input neurons in the neural network model and the remaining architecture was comprised of nine neurons in the hidden layer and one output (MLP 14-9-1).

### 2.2. Performance and Predictive Capacity of the Model

**Figure 1:**
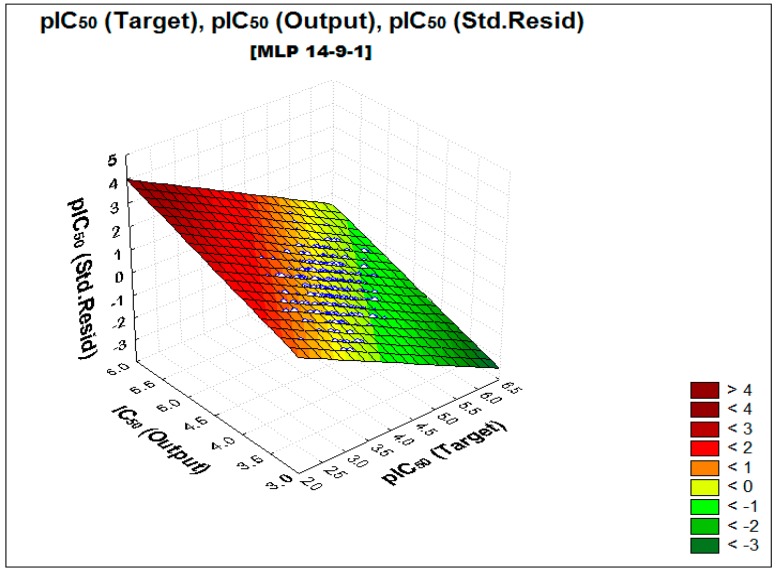
Regression plane of relation between targets, output and standard residuals values of the analyzed variable (pIC_50_).

**Figure 2:**
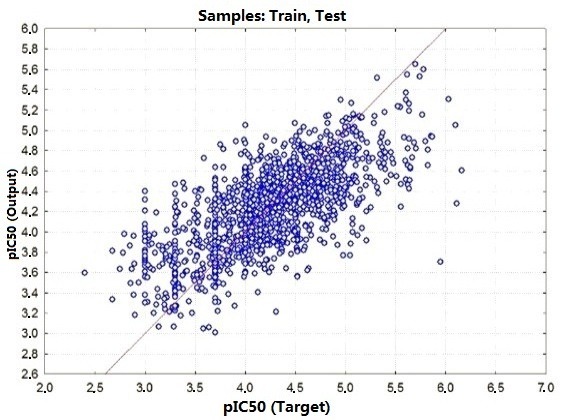
Correlation between experimental and predicted pIC_50._

The neural MLP model was trained and posteriorly validated over the test set. The correlation coefficient for the training set was $R^{2}=0.713$, and for test set it was $Q_{\text{ext}}^{2}=0.654$. The relation between the targets, output and standard residuals pIC_50_ values on the MLP model for the studied compounds is shown in Figure 1 below; the correlation between the experimental and predicted values for the training and test samples is shown in Figure 2. The whole pIC_50_ target and predicted values are available in Supporting Information (Tables S1 and S2).

### 2.3. Relative Importance of the Variables in the Model

**Figure 3:**
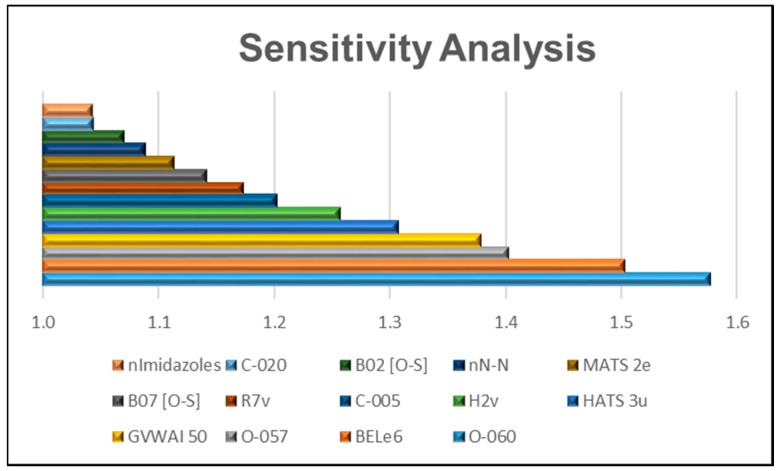
Sensitivity analysis of the MD for the MLP 14-9-1 model.

Sensitivity analysis (SA): In order to determine the relative importance of the MD used as variables in the ANN model, SA was performed. Sensitivity is a statistical parameter measured as the difference between standard deviation (SDE) values when each MD is considered as an input (SDE(n)), and when the same MD is excluded (SDE(n-1)), with both values computed over the same dataset. Greater differences are associated with higher relevance for the excluded MD [17]. Figure 3 shows the SA for the 14 MD employed in the construction of the MLP 14-9-1 network model.

**Figure 4:**
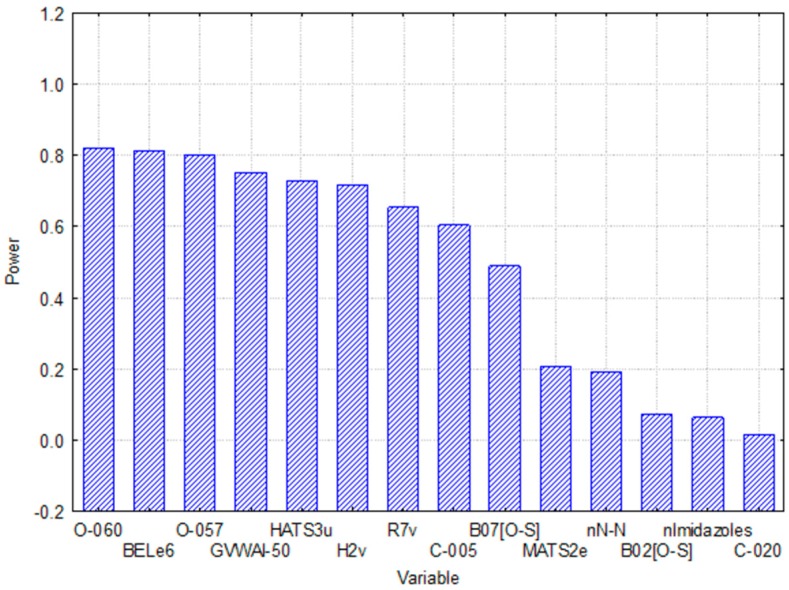
Diagram of variable importance according to the PCA.

Principal component analysis (PCA): The variable importance was also explored with a PCA using STATISTICA 8.0 software (StatSoft Inc., Tulsa, OK, USA) [17]. This method offers information about the significance of the variables and their correlation which can be used as a diagnostic tool for model interpretation. The results are shown in Figure 4 below.

### 2.4. Prediction of Coumarin Derivatives Scavenging

The MLP model was used to predict the DPPH• scavenging capacity of coumarin derivatives. Several studies have suggested that these chemical compounds possess the appropriate characteristics for potential antioxidant activity [8,9,10,11]. However, bearing in mind that any model may only appropriately predict the behavior of data points in its applicability domain (AD), the inclusion of these compounds in the model’s chemical space was assessed using the Ambit Discovery software (Nina Jeliazkova, Sofia, Bulgaria) [18]. With this analysis, the coumarin-type compounds were confirmed to lie in the network’s AD. The predicted results (expressed as pIC_50_ values) for each molecule are depicted in Table 1.

### 2.5. In Vitro Assay

The *in vitro* study of 4-hydroxycoumarin (compound **15**) was developed as an experimental corroboration of the predicted value. The pIC_50_ experimental result attained, according to the method described in Section 4.3, was 3.443.

## 3. Discussion

### 3.1. Database and Neural Network

The database of 1373 compounds with their corresponding DPPH• free radical activity values is, to the best of the authors’ knowledge, the most diverse and largest that has been reported until this moment, and it will allow for deeper study of the structure-antiradical activity relationships of chemical compounds.

The calibration ($R^{2}$) and external validation ($Q_{\text{ext}}^{2}$) values (*i.e.*, 0.713 and 0.654, respectively) of the MLP model are above the limits established for model acceptance [19], and thus indicate the satisfactory fitness and predictive capacity of the obtained model.

### 3.2. Analysis of the Molecular Descriptors

The analysis of the relative importance and a mechanistic interpretation of the 14 variables included in the MLP model provide a deeper understanding of the chemical information codified and its relationship with the modeled property. As may be observed in Figure 3 and Figure 4, the results obtained in the analysis of the relative importance of each MD based on the SA and PCA methods, respectively, exhibit great similarity. A more detailed analysis of these variables revealed the following:

- All the one-dimensional (1D) molecular descriptors included in the 14 variables, *O-060*, *O-057*, *C-005*, *C-020*, *nN-N*, *n-imidazoles*, are “Atom-centered fragments” or “Functional group counts” descriptors based in functional groups with the presence of some electronegative atom (O, N, S, Se, halogens) [20].
- On the other hand, 2D molecular descriptors are related with the presence or absence of the O-S bond (in the case of B02 (O-S) and B07 (O-S)) or, such as MATS2e y BELe6, described properties correlated with Sanderson atomic electronegativity [20].
- The 3D selected MD (HATS3u, H2v y R7v) are GATEWAY descriptors weighted by atomic van der Waals volumes [20].
- The GVWAI-50 is a drug-like molecular properties descriptor, and its values are provided only for compounds having C, H, O, N, S, Se, P, B, Si, and halogens [20].

The chemical interpretation on the importance of these MD may be retrieved from the analysis of the two currently accepted mechanisms for antioxidant activity [21], specifically to deactivate a free radical. One of them is the H-atom transfer, in which the radical reaction chain is interrupted. The intermediate molecule in this sequence of reactions has to be stable, a condition that may be obtained in molecules with electron-donating heteroatoms, or high electronic delocalization. The other mechanism is the electron transfer, in which the radical cation is first formed, followed by rapid and reversible deprotonation in solution; however, if the radical cation formed and has a sufficient lifetime, it can attack biologic molecules, even causing mutagenic effects. In both mechanisms, the presence of high electronegative atoms contributes positively to the neutralization of intermediate species, thus preventing cellular damage [22,23,24,25,26,27,28,29]. Additionally, weighting the atoms according to their chemical environment allows us to evaluate the capacity of the molecules to interact with DPPH•, and thus their scavenging capacity.

### 3.3. Prediction of Antioxidant Activities of a Group of Coumarins

Recent advances in drug discovery have resulted in an increase in the number of synthetic and naturally occurring molecules available for testing using *in vitro* assays for the scavenging ability of the DPPH• radical. Virtual screening allows for prior assessment of the potential bioactivity of chemical compounds, and thus providing key guidelines in posterior experimental work [30,31].

Coumarins form a large class of phenolic compounds occurring in plants [32]. There are numerous research initiatives aimed at studying the effects of coumarins with several positions of the hydroxyl groups and other substitutions on the scavenging activity of different radicals, including DPPH• [22,23,24,25,26]. The series of coumarin-type compounds used in this study may be divided for analysis into two groups, according to the structural analogy: Cy-analog(Compounds 1–7): *Cyclocoumarol analogous* and Wf-analog(Compounds 8–14): *Warfarine analogous*.

The DPPH• scavenging capacity predictions for the group of coumarin derivatives using the MLP model constitute the first approximation on the degree of possible antioxidant activity for this group of compounds.

The two groups of compounds have significantly different values of pIC_50_, as can be observed in Table 1. Wf-analogs clearly seem to be less effective in DPPH• radical capturing because their values of pIC_50_ were much lower (below 2.4). The non-substituted compound 4-hydroxycoumarin (compound **15**) showed an intermediate value of pIC_50_ (3.421). On the other hand, the pIC_50_ Cy-analog values were much higher (over 3.8). These results indicated a superior ability of cyclocoumarol derivatives for scavenging the DPPH• radical. Nevertheless, a more detailed analysis of the structural features is needed.

### 3.4. In Vitro Assay

The *in vitro* corroboration of the MLP model prediction showed satisfactory proximity between the experimental and predicted pIC_50_ values (*i.e.*, 3.443 and 3.421, respectively). Therefore, the built MLP model may reliably be applied in the search for new antioxidant compounds.

## 4. Materials and Methods

### 4.1. Preparation of Cases and Variables

The results of the experimentally determined scavenging ability of the DPPH• radical, (expressed in terms of the IC_50_ values) for 1373 molecules extracted from 181 scientific reports in the literature (See Supplementary Information, Table S3) produced a comprehensive and diverse dataset of compounds for the posterior analysis. All the structures were optimized using CORINA software (Molecular Networks GmbH: Erlangen, Germany) [15], and STANDARDIZER software available in the ChemAxon package (ChemAxon Ltd., Budapest, Hungary) [33]. Outlier diagnosis of the modeled compounds was performed in different moments of the study.

Furthermore, the dataset was rationally divided into training and test sets using hierarchical and *K-Means* clustering methods available in the STATISTICA 8.0 software (StatSoft Inc., Tulsa, OK, USA) [17]. Complete linkage was employed as the linkage rule and squared Euclidean distance as distance measure in the case of the former, while the optimal number of clusters for the latter were determined from the amalgamation schedule of obtained joining tree.

The parameterization of the structures was performed using 3224 molecular descriptors implemented in the DRAGON 5.5 software (TALETE srl, Milano, Italy) [34]. The correlation filter of Dragon software was applied to reduce the number of variables. Additionally, multiple linear regression coupled with the genetic algorithm in MobyDigs software (TALETE srl, Milano, Italy) [34], was employed to select the final subset of variables used in the ANN building.

The coumarin derivatives were analyzed with the Ambit Discovery software (Nina Jeliazkova, Sofia, Bulgaria) [18] to assess if they were included in the applicability domain of the MLP model, and were later optimized, and parameterized with the molecular descriptors contained in the built model.

### 4.2. Development of ANN Model

The QSAR model was developed as a Multilayer Perceptron Neural Network using STATISTICA 8.0 software (StatSoft Inc., Tulsa, OK, USA) [18]. The response variable values from the scavenging ability of the molecules (IC_50_) were transformed to their corresponding pIC_50_ values (−log IC_50_).

### 4.3. In Vitro DPPH• Assay

The free radical scavenging activity of the 4-hydroxycoumarin was measured using the stable DPPH• radical, according to Blois’s method [35]. Briefly, 3 mL of each sample solution was prepared in methanol at different concentrations (150–750 µg/mL) and was added, individually, 1 mL of DPPH• solution (0.1 mM). The mixture was shaken vigorously and left in the dark for 30 min. Then, the absorbance was measured in a Spectrophotometer (Thermo Scientific™ GENESYS 10S UV-Vis, Waltham, MA, USA) at 517 nm. BHT was used as reference in the experimental assay. This procedure was repeated three times for reproducibility. The capability to scavenge the DPPH• radical was expressed as IC_50_ (concentration of antioxidant that produces 50% of absorbance inhibition).

## 5. Conclusions

The scavenging capacity of the DPPH• radical is one of the most common methods for evaluating *in vitro* antiradical activity. An MLP neural network model was constructed to relate the structure of 1373 molecules with their scavenging activity. This model was validated using both internal and external validation techniques, showing a good predictive ability. The constructed network was used to predict the antioxidant activity of a set of coumarin-type compounds. An *in vitro* assay to further validate the predictive capacity of the built model demonstrated satisfactory proximity between experimental and predicted values, thus corroborating the performance of the model.

## Figures and Tables

**Table 1:** Predictions of the pIC_50_ values.

N^°^	Structures	IUPAC Name	Predicted pIC_50_
**1**	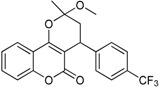	4-(4-(trifluoromethyl)phenyl)-3,4-dihydro-2-methoxy-2-methylpyrano[3,2-*c*]chromen-5(2*H*)-one	3.881
**2**	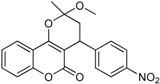	3,4-dihydro-2-methoxy-2-methyl-4-(4-nitrophenyl)pyrano[3,2-*c*]chromen-5(*2H*)-one	3.951
**3**	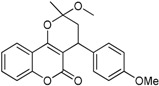	3,4-dihydro-2-methoxy-4-(4-methoxyphenyl)-2-methylpyrano[3,2-*c*]chromen-5(2*H*)-one	3.829
**4**	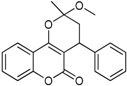	3,4-dihydro-2-methoxy-2-methyl-4-phenylpyrano[3,2-*c*]chromen-5(2*H*)-one	3.944
**5**	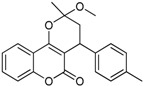	3,4-dihydro-2-methoxy-2-methyl-4-*p*-tolylpyrano[3,2-*c*]chromen-5(2*H*)-one	3.946
**6**	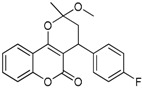	4-(4-fluorophenyl)-3,4-dihydro-2-methoxy-2-methylpyrano[3,2-*c*]chromen-5(2*H*)-one	3.959
**7**	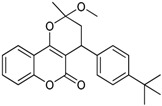	4-(4-*tert*-butylphenyl)-3,4-dihydro-2-methoxy-2-methylpyrano[3,2-*c*]chromen-5(2*H*)-one	3.903
**8**	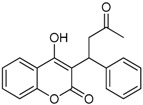	4-hydroxy-3-(3-oxo-1-phenylbutyl)-2*H*-chromen-2-one	2.326
**9**	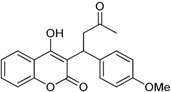	4-hydroxy-3-(1-(4-methoxyphenyl)-3-oxobutyl)-2*H*-chromen-2-one	2.291
**10**	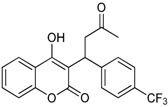	3-(1-(4-(trifluoromethyl)phenyl)-3-oxobutyl)-4-hydroxy-2*H*-chromen-2-one	2.217
**11**	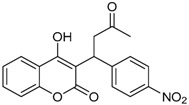	4-hydroxy-3-(1-(4-nitrophenyl)-3-oxobutyl)-2*H*-chromen-2-one	2.288
**12**	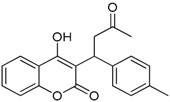	4-hydroxy-3-(3-oxo-1-p-tolylbutyl)-2*H*-chromen-2-one	2.318
**13**	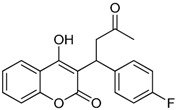	3-(1-(4-fluorophenyl)-3-oxobutyl)-4-hydroxy-2*H*-chromen-2-one	2.341
**14**	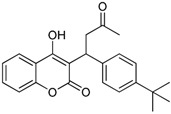	3-(1-(4-tert-butylphenyl)-3-oxobutyl)-4-hydroxy-2*H*-chromen-2-one	2.179
**15**	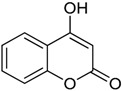	4-hydroxy-2*H*-chromen-2-one	3.421

## Supplementary Materials

Supplementary materials can be found at http://www.mdpi.com/1422-0067/17/6/881/s1.
